# Computational and Pharmacological Evaluation of Ferrocene-Based Acyl Ureas and Homoleptic Cadmium Carboxylate Derivatives for Anti-diabetic Potential

**DOI:** 10.3389/fphar.2017.01001

**Published:** 2018-01-17

**Authors:** Shahar Bano, Arif-ullah Khan, Faiza Asghar, Muhammad Usman, Amin Badshah, Saqib Ali

**Affiliations:** ^1^Riphah Institute of Pharmaceutical Sciences, Riphah International University, Islamabad, Pakistan; ^2^Department of Chemistry, Quaid-e-Azam University, Islamabad, Pakistan; ^3^Department of Chemistry, University of Wah, Wah, Pakistan

**Keywords:** ferrocene-based acyl ureas, homoleptic cadmium carboxylates, molecular docking, anti-diabetic, mice

## Abstract

We investigated possible anti-diabetic effect of ferrocene-based acyl ureas: 4-ferrocenyl aniline (PFA), 1-(4-chlorobenzoyl)-3-(4-ferrocenylphenyl) urea (DPC1), 1-(3-chlorobenzoyl)-3-(4-ferrocenylphenyl) urea (DMC1), 1-(2-chlorobenzoyl)-3-(4-ferrocenylphenyl) urea (DOC1) and homoleptic cadmium carboxylates: bis (diphenylacetato) cadmium (II) (DPAA), bis (4-chlorophenylacetato) cadmium (II) (CPAA), using *in silico* and *in vivo* techniques. PFA, DPC1, DMC1, DOC1, DPAA and CPAA exhibited high binding affinities (ACE ≥ −350 Kcal/mol) against targets: aldose reductase, peroxisome proliferator-activated receptor γ, 11β-hydroxysteroid dehydrogenase-1, C-alpha glucosidase and glucokinase, while showed moderate affinities (ACE ≥ −250 Kcal/mol) against N-alpha glucosidase, dipeptidyl peptidase-IV, phosphorylated-Akt, glycogen synthase kinase-3β, fructose-1,6-bisphosphatase and phosphoenolpyruvate carboxykinase, whereas revealed lower affinities (ACE < −250 Kcal/mol) vs. alpha amylase, protein tyrosine phosphatases 1B, glycogen phosphorylase and phosphatidylinositol 3 kinase. In alloxan (300 mg/Kg)-induced diabetic mice, DPAA and DPC1 (1–10 mg/Kg) at day 1, 5, 10, 15, and 20th decreased blood glucose levels, compared to diabetic control group and improved the treated animals body weight. DPAA (10 mg/Kg) and DPC1 (5 mg/Kg) in time-dependent manner (30–120 min.) enhanced tolerance of oral glucose overload in mice. DPAA and DPCI dose-dependently at 1, 5, and 10 mg/Kg decreased glycosylated hemoglobin levels in diabetic animals, as caused by metformin. These results indicate that aforementioned derivatives of ferrocene and cadmium possess anti-diabetic potential.

## Introduction

Diabetes mellitus (DM) is foremost health disorder, growing frequently in developing countries. Factors responsible for DM include increased deskbound lifestyle, nutrition changeover and rapid urbanization leading to widespread, parallel to rise in obesity (Hu, 2011). According to WHO, the 7th chief cause of deaths in 2030 will be DM (Mathers and Loncar, 2006). DM is mainly characterized by high blood glucose levels, i.e., hyperglycemia with altered metabolism of carbohydrates, proteins and fats due to reduced insulin secretions or/and insulin action. Type I diabetes is associated with deficiency of insulin due to autoimmune-mediated β cells damage (Tuomi, 2005). Hyperglycemic condition in type I diabetes is controlled by administering exogenous insulin via subcutaneous route. However, type II diabetes is associated with relatively reduced levels or/and reduced sensitivity of hepatic, cardiac and fat cell toward insulin action. Thus, patients with type II diabetes rely on synthetic anti-diabetic therapy (Nathan et al., 2009).

Until now, many of the metals have been reported to possess anti-diabetic potential, such as vanadium (Heyliger et al., 1985), chromium (Anderson et al., 1997), cobalt (Ybarra et al., 1997), molybdenum (Ozcelikay et al., 1996), tungsten (Barberà et al., 2001), cadmium (Gümüşlü et al., 1997), iron, and copper (Siva and Kumar, 2013). Ferrocene derivatives containing iron moiety have been reported for good binding affinity to DNA, having cytotoxic, anticancer, antimalarial, antibiotic and antiviral activities (Lal et al., 2011; Asghar et al., 2017). Acyl urea group possesses anticancer, anticonvulsant, antimicrobial and antioxidant activities. Acyl urea derivatives have also been reported for anti-diabetic effect by inhibition of human liver glycogen phosphorylase (Klabunde et al., 2005). High levels of selenium in serum are associated with DM prevalence (Bleys et al., 2007). Cadmium is responsible for decrease in plasma selenium levels in alloxane treated diabetic rats (Gümüşlü et al., 1997). Carboxylate complexes with tin have been reported for α-glucosidase inhibition (Roy et al., 2015).

**Figure 1:**
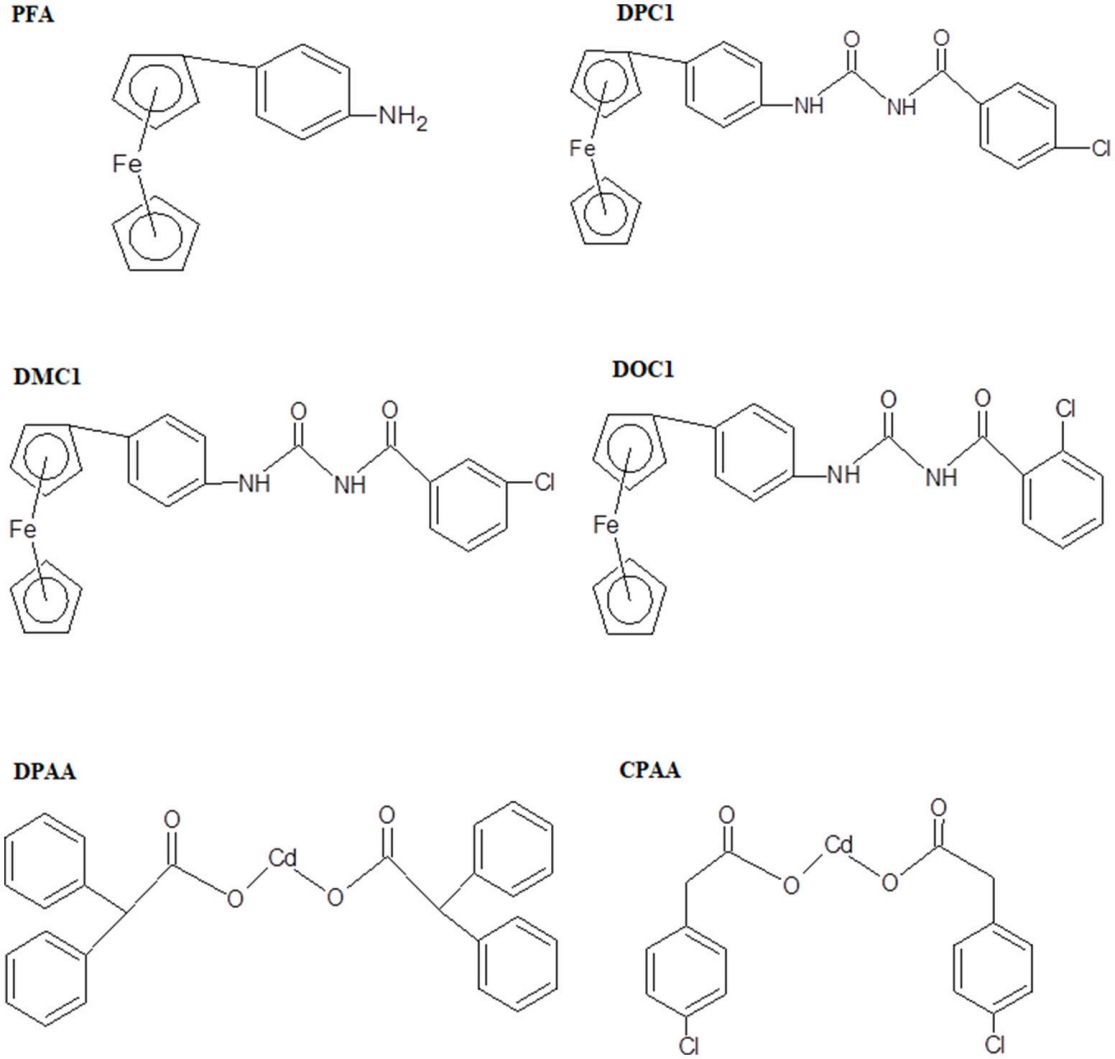
Chemical structures of ferrocene incorporated acyl ureas and homoleptic cadmium carboxylates: 4-ferrocenyl aniline (PFA), 1-(4-chlorobenzoyl)-3-(4-ferrocenylphenyl) urea (DPC1), 1-(3-chlorobenzoyl)-3-(4-ferrocenylphenyl) urea (DMC1), 1-(2-chlorobenzoyl)-3-(4-ferrocenylphenyl) urea (DOC1), bis (diphenylacetato) cadmium (II) (DPAA), and bis (4-chlorophenylacetato) cadmium (II) (CPAA), drawn through Chem. Sketch 2015 2.5 software.

In present study, we made an effort to explore anti-diabetic potential of ferrocene and cadmium selected derivatives including 4-ferrocenyl aniline (PFA), 1-(4-chlorobenzoyl)-3-(4-ferrocenylphenyl) urea (DPC1), 1-(3-chlorobenzoyl)-3-(4-ferrocenylphenyl) urea (DMC1), 1-(2-chlorobenzoyl)-3-(4-ferrocenylphenyl) urea (DOC1), bis (diphenylacetato) cadmium (II) (DPAA), and bis (4-chlorophenylacetato) cadmium (II) (CPAA), through molecular docking and *in vivo* animals experimentation models. The chemical structures of test compounds were drawn via Chem. Sketch 2015 2.5 (Figure 1).

## Materials and methods

### Chemicals

Alloxan monohydrate and dimethyl sulphoxide (DMSO) were purchased from Sigma-Aldrich Co. LLC, U.S.A. Metformin HCL was obtained from Caraway Pharmaceuticals, National Industrial Zone Rawat, Islamabad, Pakistan. Ferrocene incorporated acyl ureas and homoleptic cadmium carboxylates were gifted by the Department of Chemistry, Quaid-e-Azam University. All chemicals were of analytical grade.

### Animals

Adult Balb-C mice of either sex were kept under controlled temperature (22–25°C) at the animal house of Riphah Institute of Pharmaceutical Sciences, Islamabad, Pakistan. Animals were given free access to standard diet and water *ad libitum*. Experiments performed complied with rulings of Institute of Laboratory Animal Resources, Commission on Life Sciences University, National Research Council (1996), approved by Ethical Committee, Riphah Institute of Pharmaceutical Sciences (Ref. No.: REC/RIPS/2016/0013).

### Docking studies

**Figure 2:**
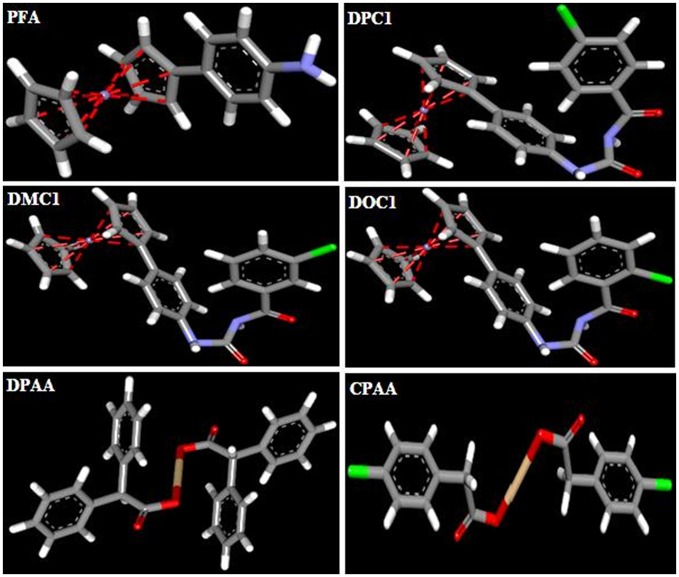
3D-structures of ferrocene incorporated acyl ureas and homoleptic cadmium carboxylates: 4-ferrocenyl aniline (PFA), 1-(4-chlorobenzoyl)-3-(4-ferrocenylphenyl) urea (DPC1), 1-(3-chlorobenzoyl)-3-(4-ferrocenylphenyl) urea (DMC1), 1-(2-chlorobenzoyl)-3-(4-ferrocenylphenyl) urea (DOC1), bis (diphenylacetato) cadmium (II) (DPAA), and bis (4-chlorophenylacetato) cadmium (II) (CPAA), drawn through Guass View 5.0 Software and saved into PDB format. Atoms are shown by colors; gray color (carbon atoms), white color (hydrogen atoms), red color (oxygen atoms), blue color (nitrogen atoms), yellowish color (cadmium atoms), and green color (chlorine atoms).

**Figure 3:**
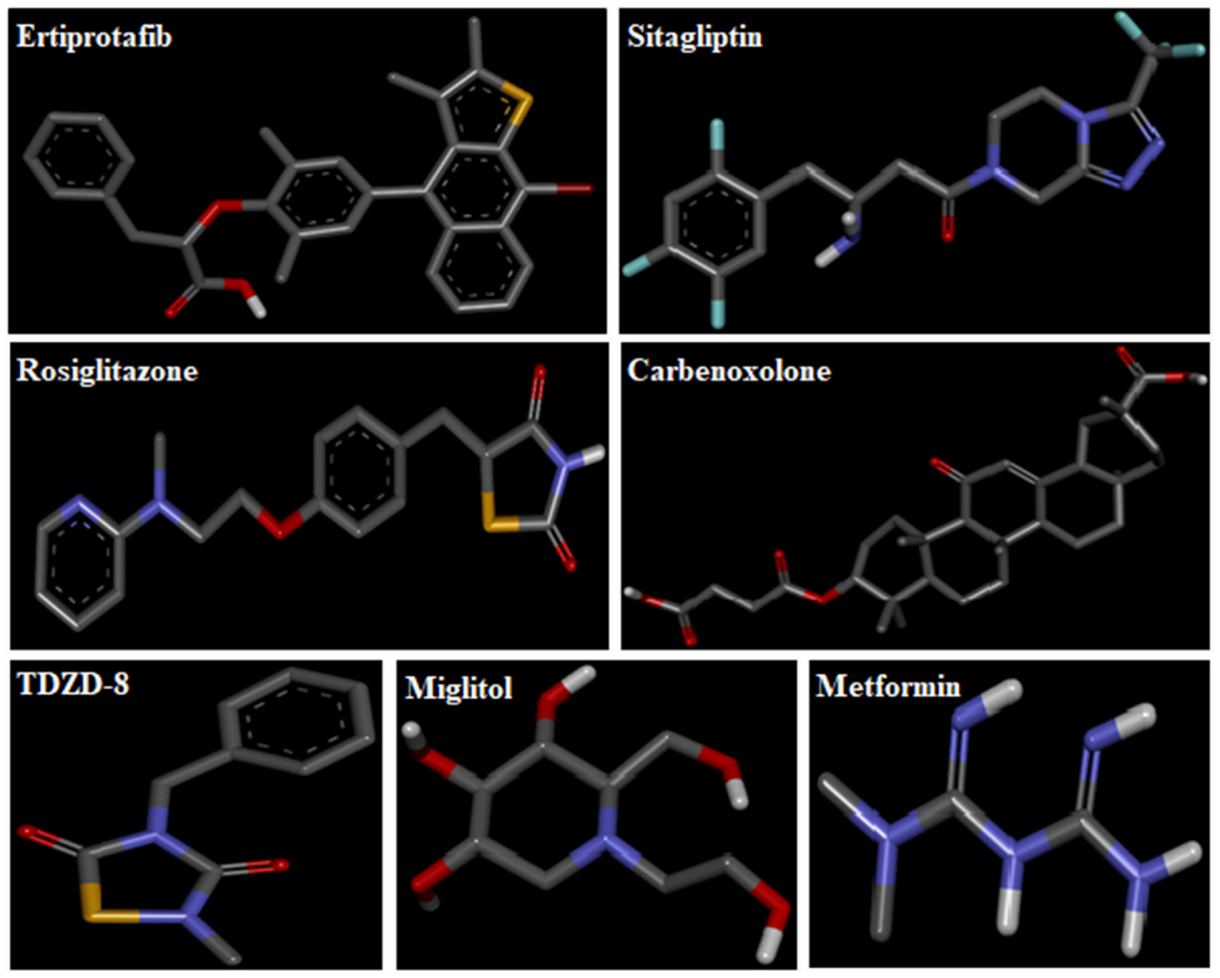
3D-structures of reference drugs: miglitol, metformin, carbenoxolone, thiadiazolidinone-8 (TDZD-8), rosiglitazone, sitagliptin and ertiprotafib, drawn through Chem. Sketch 2015 2.5 and saved in PDB format through Biovia Discovery Studio 2016. Atoms are shown by colors; gray color (carbon atoms), white color (hydrogen atoms), red color (oxygen atoms), blue color (nitrogen atoms), dark red (bromine), and yellow color (sulfur atoms).

**Figure 4:**
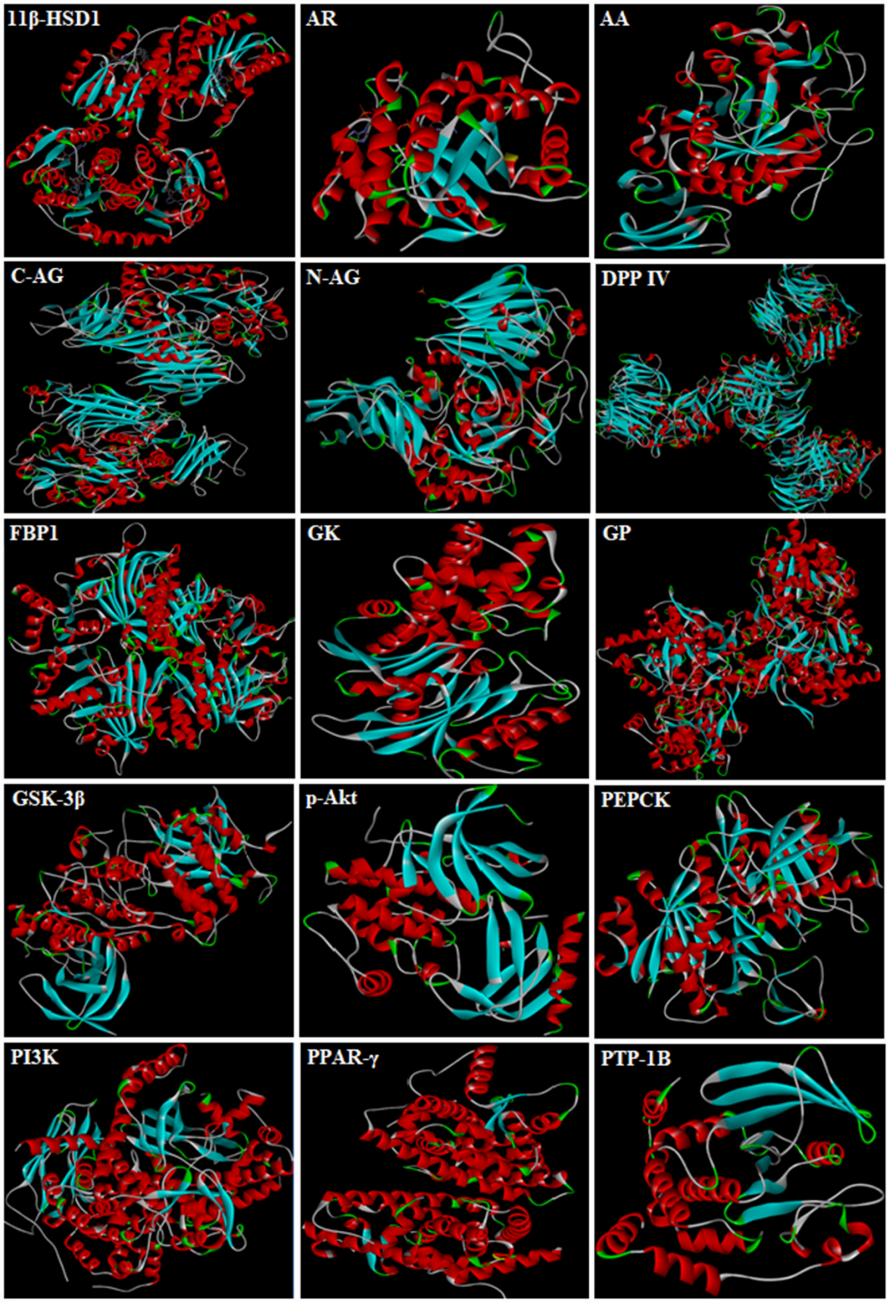
3D-structures of protein targets involved in diabetes: alpha amylase (AA), C-alpha glucosidase (C-AG), N-alpha glucosidase (N-AG), aldose reductase (AR), glucokinase (GK), glycogen phosphorylase (GP), fructose-1,6-bisphosphatase (FBP1), phosphoenolpyruvate carboxykinase (PEPCK), 11β-hydroxysteroid dehydrogenase-1 (11β-HSD1), glycogen synthase kinase-3β (GSK-3β), peroxisome proliferator-activated receptor γ (PPAR-γ), phosphatidylinositol 3 kinase (PI3K), phosphorylated-Akt (p-Akt), dipeptidyl peptidase-IV (DPP IV) and protein tyrosine phosphatase 1B (PTP-1B).

3D-structures of the test compounds (PFA, DPC1, DMC1, DOC1, DPAA, and CPAA) were formed through Gauss View 5.0 software (Figure 2). 3D-structures of standard drug were obtained by converting 2D-structures through Biovia Discovery Studio Visualizer (DSV) 2016 (Figure 3). Polar hydrogen atoms (H-atoms) were added through same software, followed by saving into PDB format. Standard drugs were: miglitol, metformin, carbenoxolone, thiadiazolidinone-8 (TDZD-8), rosiglitazone, sitagliptin and ertiprotafib. 3D-structures of human protein targets involved in DM were retrieved from online data bank, RCSB PDB (https://www.rcsb.org/pdb/), as shown in Figure 4, according to their PDB IDs (Sussman et al., 1998). Target proteins were: alpha amylase (AA, PDB ID: 2QMK), C-alpha glucosidase (C-AG, PDB ID: 3TON), N-alpha glucosidase (N-AG, PDB ID: 2QMJ), aldose reductase (AR, PDB ID: 1US0), glucokinase (GK, PDB ID: IV4S), glycogen phosphorylase (GP, PDB ID: 1L7X), fructose-1,6-bisphosphatase (FBP1, PDB ID: 2JJK), phosphoenolpyruvate carboxykinase (PEPCK, PDB ID: 1KHB), 11β-hydroxysteroid dehydrogenase-1 (11β-HSD1, PDB ID: 2BEL), glycogen synthase kinase-3β (GSK-3β, PDB ID: 1Q4L), peroxisome proliferator-activated receptor γ (PPAR-γ, PDB ID: 2PRG), phosphatidylinositol 3 kinase (PI3K, PDB ID: 1E7U), phosphorylated-Akt (p-Akt, PDB ID: 3O96), dipeptidyl peptidase-IV (DPP IV, PDB ID: 2ONC), and protein tyrosine phosphatase 1B (PTP-1B, PDB ID: 2F70). By using same software, water molecules and ligands were removed and polar H-atoms were added, followed by saving in PDB format. Molecular docking was performed by PatchDock server, which is an online, geometry based automatic docking tool (Duhovny et al., 2002). We selected Root Mean Square Deviation clustering value at 2.0 to discard the redundant solutions of docking. Docking was executed and evaluated on bases of atomic contact energy (ACE) value (Kcal/mol) (Schneidman-Duhovny et al., 2005). Top 20 poses were evaluated and one with lowest ACE value (Kcal/mol) was selected for evaluation through Biovia DSV 2016. Each complex was assessed in 3D pattern to check the maximum binding interactions formed between ligands and amino acid residues including: alanine (ALA), arginine (ARG), asparagine (ASN), aspartic acid (ASP), cysteine (CYS), glutamine (GLN), glutamic acid (GLU), glycine (GLY), histidine (HIS), isoleucine (ILE), lysine (LYS), methionine (MET), phenylalanine (PHE), proline (PRO), serine (SER), threonine (THR), tryptophan (TRP), tyrosine (TYR), threonine (THR), and valine (VAL).

### Blood glucose levels and body weight measurement

Balb-C mice were adapted to the laboratory conditions and kept on overnight fasting (12–14 h). Alloxan was used for the induction of diabetes (Dunn and McLetchie, 1943). Solution of alloxan monohydrate (300 mg/Kg) was freshly prepared in saline and injected intra-peritoneally to mice (Bukhari et al., 2015). After 48 h, tail prick method was used to measure blood glucose levels of animals. Mice with blood glucose levels ≥200 mg/dL were considered as diabetic (Saudek et al., 2008). On basis of docking results, two most potential compounds were selected for *in vivo* studies. For DPAA and DPC1, animals were placed in six groups for each compound. The sample size in each group comprised of five mice. Group I and II were non-diabetic control and diabetic control, injected with saline (10 mL/Kg) and alloxan monohydrate (300 mg/Kg) respectively. Group III, IV, and V were alloxan-induced diabetic mice, administered with the test compound at doses of 1, 5, and 10 mg/Kg respectively. Group VI was positive control and injected with metformin (500 mg/Kg). Blood glucose levels were measured at day 1, 5, 10, 15, and 20th, using Easy Gluco auto-coding glucometer. For complete treatment period, body weight of animals was measured at same regular intervals.

### Oral glucose tolerance test (OGTT)

After keeping on 18 h fasting, mice were placed into four groups (for both compounds total eight groups). The sample size in each group comprised of five mice. Group I and II were non-diabetic and diabetic control, injected with saline (10 mL/Kg) and alloxan (300 mg/Kg) respectively. Group III was treated with the test compound (DPAA, 10 mg/Kg/DPC1, 5 mg/Kg). Group IV was positive control and injected with metformin (500 mg/Kg). All groups were pre-treated and after 30 min. D-glucose load of 2 g/Kg was given orally. Blood glucose levels were measured at 0, 30, 60, 90, and 120 min., using Easy Gluco auto-coding glucometer (Marguet et al., 2000).

### Glycosylated hemoglobin (HbA1C) test

After 6 weeks of treatment, HbA1C test was performed (Asgary et al., 2008) for all groups having sample size in each group comprised of five mice. Blood samples were collected using cardiac puncture method (Doeing et al., 2003). HbA1C level test carried out in Cantt Laboratory and Medical Imaging, Rawalpindi.

### Acute toxicity test

Animals were kept on overnight fasting and distributed into different groups for each dose of DPAA (15, 25, 50, and 100 mg/Kg) and DPC1 (25, 50, and 100 mg/Kg). The sample size in each group comprised of five mice. After administration of compounds, animals were kept under observation for 7 days to determine mortality (Chen et al., 2009).

### Statistical analysis

Data expressed as mean ± standard error of mean (SEM). Significance of results was assessed by one-way analysis of variance (ANOVA), followed by *post-hoc* Tukey's test. *P* < 0.05 was deliberated to be statistically significant. The statistical assessment, preparation of graphs and evaluation was performed by using Graph Pad Prism 5.01.

## Results

### Docking evaluation

Docking evaluation was done by assessing ACE values, number of hydrogen bonds (H-bonds), number of π-π bonds and hydrophobic interactions formed between ligand-protein complexes. ACE (Kcal/mol) values for complexes of ligands and targets are shown in Table 1. Number of H-bonds and amino acids involved in making H-bonds are presented in Table 2. Number of π-π bonds and amino acids forming π-π bonds are expressed in Table 3. Hydrophobic interactions of best docked poses for ligand-protein complexes are plotted in Table 4. Interactions formed by DPAA, CPAA, PFA, DPC1, DMC1, DOC1 and standard drugs against AA, C-AG, N-AG, AR, GK, GP, FBP1, PEPCK, 11β-HSD1, GSK-3β, PPAR-γ, PI3K, p-Akt, DPP IV, and PTP-1B are shown in Figures S1–S15, respectively.

**Table 1:** ACE values (Kcal/mol) of best docked poses of 4-ferrocenyl aniline (PFA), 1-(4-chlorobenzoyl)-3-(4-ferrocenylphenyl) urea (DPC1), 1-(3-chlorobenzoyl)-3-(4-ferrocenylphenyl) urea (DMC1), 1-(2-chlorobenzoyl)-3-(4-ferrocenylphenyl) urea (DOC1), bis (diphenylacetato) cadmium (II) (DPAA), bis (4-chlorophenylacetato) cadmium (II) (CPAA) and standard drugs against alpha amylase (AA), C-alpha glucosidase (C-AG), N-alpha glucosidase (N-AG), aldose reductase (AR), glucokinase (GK), glycogen phosphorylase (GP), fructose-1,6-bisphosphatase (FBP1), phosphoenolpyruvate carboxykinase (PEPCK), 11β-hydroxysteroid dehydrogenase-1 (11β-HSD1), glycogen synthase kinase-3β (GSK-3β), peroxisome proliferator-activated receptor γ (PPAR-γ), phosphatidylinositol 3 kinase (PI3K), phosphorylated-Akt (p-Akt), dipeptidyl peptidase-IV (DPP IV) and protein tyrosine phosphatase 1B (PTP-1B).

**Target proteins**	**PDB ID**	**Binding energies (ACE values Kcal/mol)**						
		**PFA**	**DPC1**	**DMC1**	**DOC1**	**DPAA**	**CPAA**	**Standard drugs**
AA	2QMK	−177.77	−236.47	−231.29	−204.63	−277.18	−188.19	−131.81^A^
C-AG	3TON	−235.33	−397.72	−373.81	−374.69	−438.12	−301.76	−152.38^A^
N-AG	2QMJ	−260.19	−272.06	−320.05	−330.17	−369.10	−260.85	−249.33^A^
AR	1US0	−273.94	−383.49	−368.48	−387.05	−378.39	−286.37	−152.13^B^
GK	IV4S	−298.70	−426.22	−416.20	−412.65	−493.09	−299.26	−187.66^B^
GP	1L7X	−135.82	−206.36	−217.05	−243.26	−223.13	−198.71	−154.92^B^
FBP1	2JJK	−206.28	−341.22	−357.58	−344.46	−410.97	−151.99	−155.95^B^
PEPCK	1KHB	−196.02	−249.85	−302.87	−259.64	−292.61	−243.38	−152.17^B^
11β-HSD1	2BEL	−242.95	−388.87	−385.37	−377.20	−425.22	−278.48	−446.12^C^
GSK-3β	1Q4L	−204.83	−320.12	−253.96	−318.44	−343.95	−232.88	−209.66^D^
PPAR-γ	2PRG	−242.20	−361.51	−359.73	−340.37	−406.60	−273.52	−371.55^E^
PI3K	1E7U	−186.15	−195.32	−188.77	−210.48	−306.43	−254.74	−327.40^E^
p-Akt	3O96	−198.07	−272.81	−274.82	−288.05	−301.65	−176.69	−278.74^E^
DPP IV	2ONC	−133.07	−299.40	−299.99	−307.36	−273.27	−251.90	−171.61^F^
PTP-1B	2F70	−159.90	−215.85	−221.12	−147.93	−241.48	−170.28	−283.57^G^

*Standard inhibitors or activator of pathways are: (A) miglitol, (B) metformin, (C) carbenoxolone, (D) thiadiazolidinone-8, (E) rosiglitazone, (F) sitagliptin, and (G) ertiprotafib*.

**Table 2:** Hydrogen bonds (H-bonds) formed by 4-ferrocenyl aniline (PFA), 1-(4-chlorobenzoyl)-3-(4-ferrocenylphenyl) urea (DPC1), 1-(3-chlorobenzoyl)-3-(4-ferrocenylphenyl) urea (DMC1), 1-(2-chlorobenzoyl)-3-(4-ferrocenylphenyl) urea (DOC1), bis (diphenylacetato) cadmium (II) (DPAA), bis (4-chlorophenylacetato) cadmium (II) (CPAA) and standard drugs against alpha amylase (AA), C-alpha glucosidase (C-AG), N-alpha glucosidase (N-AG), aldose reductase (AR), glucokinase (GK), glycogen phosphorylase (GP), fructose-1,6-bisphosphatase (FBP1), phosphoenolpyruvate carboxykinase (PEPCK), 11β-hydroxysteroid dehydrogenase-1 (11β-HSD1), glycogen synthase kinase-3β (GSK-3β), peroxisome proliferator-activated receptor γ (PPAR-γ), phosphatidylinositol 3 kinase (PI3K), phosphorylated-Akt (p-Akt), dipeptidyl peptidase-IV (DPP IV) and protein tyrosine phosphatase 1B (PTP-1B).

**Proteins**	**PDB ID**	**PFA**		**DPC1**		**DMC1**		**DOC1**		**DPAA**		**CPAA**		**Standard drugs**	
		**H-bonds**	**Amino acids**	**H-bonds**	**Amino acids**	**H-bonds**	**Amino acids**	**H-bonds**	**Amino acids**	**H-bonds**	**Amino acids**	**H-bonds**	**Amino acids**	**H-bonds**	**Amino acids**
AA	2QMK	2	GLY304	1	ASP353	0	–	1	ASN352	1	ARG346	0	–	2^A^	ILE312
			ILE312												THR314
C-AG	3TON	0	–	1	ASN1776	3	ASN1776	1	ASN1776	0	–	2	ASN1827	0^A^	–
							VAL1812						ASN1827		
							VAL1809								
N-AG	2QMJ	0	–	2	SER120	0	–	5	ALA537	1	SER288	0	–	2^A^	GLY533
					SER120				ILE523						ALA536
									PHE535						
									ARG520						
									SER521						
AR	1US0	0	–	2	ALA299	1	CYS303	0	–	0	–	1	TRP111	1^B^	HIS110
					CYS298										
GK	IV4S	0	–	0	–	0	–	1	ARG63	2	SER64	0	–	1^B^	VAL452
											ALA456				
GP	1L7X	0	–	3	HIS377	1	HIS377	1	LYS680	3	THR676	0	–	3^B^	GLY186
					ASN484						GLY675				GLY186
					ASN484						ARG569				TYR52
FBP1	2JJK	0	–	1	ALA189	0	–	1	ALA189	0	–	1	LYS72	3^B^	GLY26
															GLY26
															MET18
PEPCK	1KHB	0	–	1	PRO337	1	VAL335	1	PHE530	1	ARG436	1	ASN292	0^B^	–
11β-HSD1	2BEL	2	NAP1278	0	–	2	THR124	0	–	1	THR222	0	–	2^C^	TYR177
			NAP1278				NAP1278								TYR177
GSK-3β	1Q4L	1	ASN64	0	–	0	–	0	–	1	ASN95	1	ARG223	0^D^	–
PPAR-γ	2PRG	1	MET348	0	–	0	–	0	–	0	–	1	SER289	0^E^	–
PI3K	1E7U	0	–	2	THR1043	1	ASP632	2	ASP632	0	–	0	–	0^E^	–
					THR1043				ASN634						
p-Akt	3O96	0	–	2	THR211	2	VAL271	0	–	0	–	0	–	1^E^	ILE290
					THR211		ASN54								
DPP IV	2ONC	1	VAL121	0	–	0	–	3	PHE364	0	–	1	ASN272	3^F^	GLY99
									ALA306						ASP96
									TRP305						LYS71
PTP-1B	2F70	0	–	0	–	1	LYS73	1	ARG199	0	–	0	–	2^G^	PRO206
															HIS208

*Standard inhibitors or activator of pathways are: (A) miglitol, (B) metformin, (C) carbenoxolone, (D) thiadiazolidinone-8, (E) rosiglitazone, (F) sitagliptin and (G) ertiprotafib. Amino acids are: ALA, alanine; ARG, arginine; ASN, asparagine; ASP, aspartic acid; CYS, cysteine; GLN, glutamine; GLU, glutamic acid; GLY, glycine; HIS, histidine; ILE, isoleucine; LYS, lysine; MET, methionine; PHE, phenylalanine; PRO, proline; SER, serine; THR, threonine; TRP, tryptophan; TYR, tyrosine; VAL, valine*.

**Table 3:** Pi-Pi bonds (π-π bonds) formed by 4-ferrocenyl aniline (PFA), 1-(4-chlorobenzoyl)-3-(4-ferrocenylphenyl) urea (DPC1), 1-(3-chlorobenzoyl)-3-(4-ferrocenylphenyl) urea (DMC1), 1-(2-chlorobenzoyl)-3-(4-ferrocenylphenyl) urea (DOC1), bis (diphenylacetato) cadmium (II) (DPAA), bis (4-chlorophenylacetato) cadmium (II) (CPAA) and standard drugs against alpha amylase (AA), C-alpha glucosidase (C-AG), N-alpha glucosidase (N-AG), aldose reductase (AR), glucokinase (GK), glycogen phosphorylase (GP), fructose-1,6-bisphosphatase (FBP1), phosphoenolpyruvate carboxykinase (PEPCK), 11β-hydroxysteroid dehydrogenase-1 (11β-HSD1), glycogen synthase kinase-3β (GSK-3β), peroxisome proliferator-activated receptor γ (PPAR-γ), phosphatidylinositol 3 kinase (PI3K), phosphorylated-Akt (p-Akt), dipeptidyl peptidase-IV (DPP IV) and protein tyrosine phosphatase 1B (PTP-1B).

**Proteins**	**PDB ID**	**PFA**		**DPC1**		**DMC1**		**DOC1**		**DPAA**		**CPAA**		**Standard drugs**	
		**π-π bonds**	**Amino acids**	**π-π bonds**	**Amino acids**	**π-π bonds**	**Amino acids**	**π-π bonds**	**Amino acids**	**π-π bonds**	**Amino acids**	**π-π bonds**	**Amino acids**	**π-π bonds**	**Amino acids**
AA	2QMK	1	GLN302	1	PHE348	0	–	0	–	1	TRP316	0	–	0^A^	–
C-AG	3TON	0	–	0	–	0	–	0	–	0	–	0	–	0^A^	–
N-AG	2QMJ	2	GLY157	0	–	0	–	0	–	0	–	0	–	0^A^	–
			LYS48												
AR	1US0	2	ALA299	2	TRP20	2	TRP20	3	TRP20	0	–	0	–	0^B^	–
			TRP111		TRP111		TRP111		TRP111						
									PHE122						
GK	IV4S	0	–	1	SER64	0	–	0	–	1	HIS218	0	–	0^B^	–
GP	1L7X	0	–	0	–	0	–	0	–	0	–	0	–	0^B^	–
FBP1	2JJK	0	–	0	–	0	–	0	–	0	–	0	–	0^B^	–
PEPCK	1KHB	0	–	0	–	0	–	0	–	0	–	0	–	0^B^	–
11β-HSD1	2BEL	0	–	0	–	0	–	1	TYR183	1	TYR177	1	TYR177	0^C^	–
GSK-3β	1Q4L	0	–	0	–	0	–	0	–	0	–	0	–	0^D^	–
PPAR-γ	2PRG	0	–	0	–	0	–	0	–	0	–	0	–	0^E^	–
PI3K	1E7U	0	–	0	–	0	–	0	–	0	–	1	TYR608	0^E^	–
p-Akt	3O96	0	–	0	–	0	–	1	TRP80	0	–	1	TRP80	1^E^	TRP80
DPP IV	2ONC	0	–	0	–	0	–	0	–	1	TRP154	0	–	0^F^	–
PTP-1B	2F70	0	–	0	–	0	–	0	–	0	–	0	–	0^G^	–

*Standard inhibitors or activator of pathways are: (A) miglitol, (B) metformin, (C) carbenoxolone, (D) thiadiazolidinone-8, (E) rosiglitazone, (F) sitagliptin, and (G) ertiprotafib. Amino acids are: ALA, alanine; GLN, glutamine; GLY, glycine; HIS, histidine; LYS, lysine; PHE, phenylalanine; SER, Serine; TRP, tryptophan; TYR, tyrosine*.

**Table 4:** Hydrophobic interactions formed by 4-ferrocenyl aniline (PFA), 1-(4-chlorobenzoyl)-3-(4-ferrocenylphenyl) urea (DPC1), 1-(3-chlorobenzoyl)-3-(4-ferrocenylphenyl) urea (DMC1), 1-(2-chlorobenzoyl)-3-(4-ferrocenylphenyl) urea (DOC1), bis (diphenylacetato) cadmium (II) (DPAA), bis (4-chlorophenylacetato) cadmium (II) (CPAA) and standard drugs against alpha amylase (AA), C-alpha glucosidase (C-AG), N-alpha glucosidase (N-AG), aldose reductase (AR), glucokinase (GK), glycogen phosphorylase (GP), fructose-1,6-bisphosphatase (FBP1), phosphoenolpyruvate carboxykinase (PEPCK), 11β-hydroxysteroid dehydrogenase-1 (11β-HSD1), glycogen synthase kinase-3β (GSK-3β), peroxisome proliferator-activated receptor γ (PPAR-γ), phosphatidylinositol 3 kinase (PI3K), phosphorylated-Akt (p-Akt), dipeptidyl peptidase-IV (DPP IV) and protein tyrosine phosphatase 1B (PTP-1B).

**Protein targets**	**PDB ID**	**Amino acid residues forming hydrophobic interactions**						
		**PFA**	**DPC1**	**DMC1**	**DOC1**	**DPAA**	**CPAA**	**Standard drugs**
AA	2QMK	THR314	GLY304	TRP59	ASN352	ASN352	GLU484	^−A^
					GLY351	PHE348	SER478	
						ARG346		
C-AG	3TON	MET1778	ASN1776	VAL1809	ASN1776	ASN1776	VAL1807	^−A^
			LEU1740	SER1811	LEU1740	VAL1812	VAL1809	
			1LE1801	THR1810	ILE1801	THR1810	ASN1776	
				VAL1812	THR1810	MET1778		
				SER1813				
				1LE1814				
N-AG	2QMJ	SER155	VAL116	LYS534	ILE523	LYS776	ALA537	ALA285^A^
			SER118	ALA285	PHE522	PHE535	ALA285	
			GLN117	LYS776	ALA285	ALA285	ASP777	
			PHE119			PRO287		
			HIS115			LEU286		
AR	1US0	–	CYC298	CYS298	CYS298	TRP111	TYR48	^−B^
			TRP219		TRP79	VAL47		
						TRP219		
GK	IV4S	–	PRO66	–	VAL455	ILE211	PRO66	ARG63^B^
			TYR215		PRO66	VAL455	THR65	
			THR65		THR65	PRO66	TYR214	
						THR65		
						TYR214		
						VAL455		
GP	1L7X	GLY135	GLY135	HIS377	GLY134	GLY677	ALA265	^−B^
			LEU136	GLY135	GLY135	THR676		
			VAL455		LYS680	LEU136		
			ALA673		ARG569	ALA673		
			TYR573			HIS377		
FBP1	2JJK	–	SER46	–	SER46	ALA51	ALA51	^−B^
			ALA51		ALA51	PRO188	LYS72	
							LYS50	
PEPCK	1KHB	MN701	THR339	ASN292	ASN292	PRO337	PHE525	ASN533
		LYS290	ASN344	PRO337	PHE530	THR343	GLY289	PHE525^B^
			GLY338	THR339	THR343	PHE530		
						VAL335		
						ASN292		
11β-HSD1	2BEL	ILE121	ALA226	THR222	THR124	ALA223	LEU171	THR222
			THR124		ALA226	VAL227	THR124	THR124
						THR222	ASN123	ASN123
						SER170	THR222	TYR177^C^
GSK-3β	1Q4L	–	LEU132	ASP200	LEU188	ASP90	ARG223	ASN64^D^
						GLN295	ILE228	
						PRO294	SER215	
							ASN287	
PPAR-γ	2PRG	–	CYS285	MET364	–	GLY284	HIS449	CYS285^E^
						LEU330	CYS285	
PI3K	1E7U	–	PHE497	ASN634	PRO563	TRP229	TRP355	TRP355
			THR1043	LYS591	LEU564	SER824	ALA528	ALA528^E^
			SER1044			LEU823	ILE420	
			LYS1045			GLU826		
						ASN825		
p-Akt	3O96	VAL270	–	GLN79	–	LEU264	LEU264	THR291^E^
		ILE290				TYR272		
DPP IV	2ONC	–	–	–	–	THR156	VAL279	PHR98
						ILE107	SER277	PHE95
							THR280	GLU97
							TYR330	ASP96^F^
PTP-1B	2F70	LYS73	LYS73	LYS73	GLY202	LYS73	GLN78	GLN102
			GLN78	GLN78		GLN78	SER80	HIS208
						PRO206	HIS60	PRO206^G^

*Standard inhibitors or activator of pathways are: (A) miglitol (B) metformin, (C) carbenoxolone, (D) thiadiazolidinone-8, (E) rosiglitazone, (F) sitagliptin and (G) ertiprotafib. Amino acids are: ALA, alanine; ARG, arginine; ASN, asparagine; ASP, aspartic acid; CYS, cysteine; GLN, glutamine; GLU, glutamic acid; GLY, glycine; HIS, histidine; ILE, isoleucine; LYS, lysine; MET, methionine; PHE, phenylalanine; PRO, proline; SER, serine; THR, threonine; TRP, tryptophan; TYR, tyrosine; VAL, valine*.

### Effect on blood glucose levels

**Figure 5:**
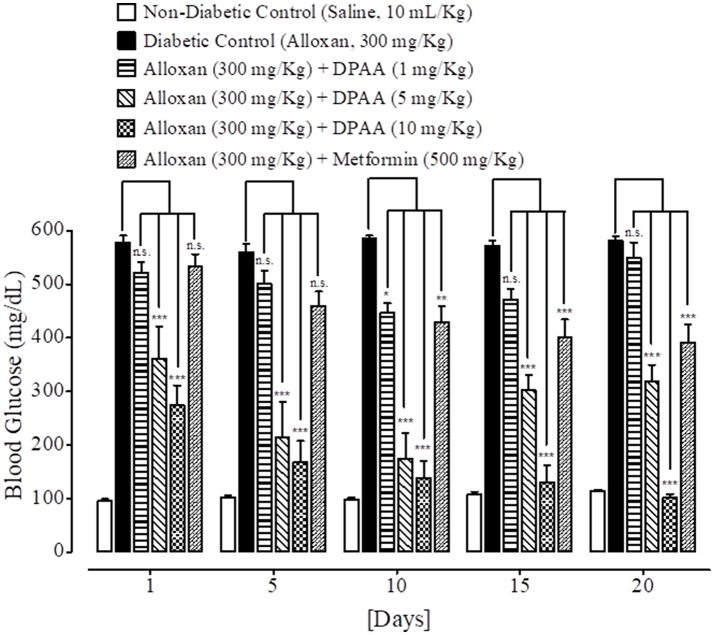
Bar-graph representing blood glucose levels at different treatment days of saline treated group (non-diabetic control), alloxan treated group (diabetic control), inhibitory effect of bis (diphenylacetato) cadmium (II) (DPAA) at different doses (1–10 mg/Kg) and metformin treated group against alloxan-induced hyperglycemia in mice. Data presented as mean ± SEM. Statistical analysis used one-way ANOVA, followed by *post-hoc* Tukey's test. ^*^P < 0.05, ^**^P < 0.01, ^***^P < 0.001 comparison of the blood glucose levels of DPAA and metformin treated groups vs. diabetic control group. n.s., non-significant. The sample size in each group comprised of five mice (*n* = 5).

**Figure 6:**
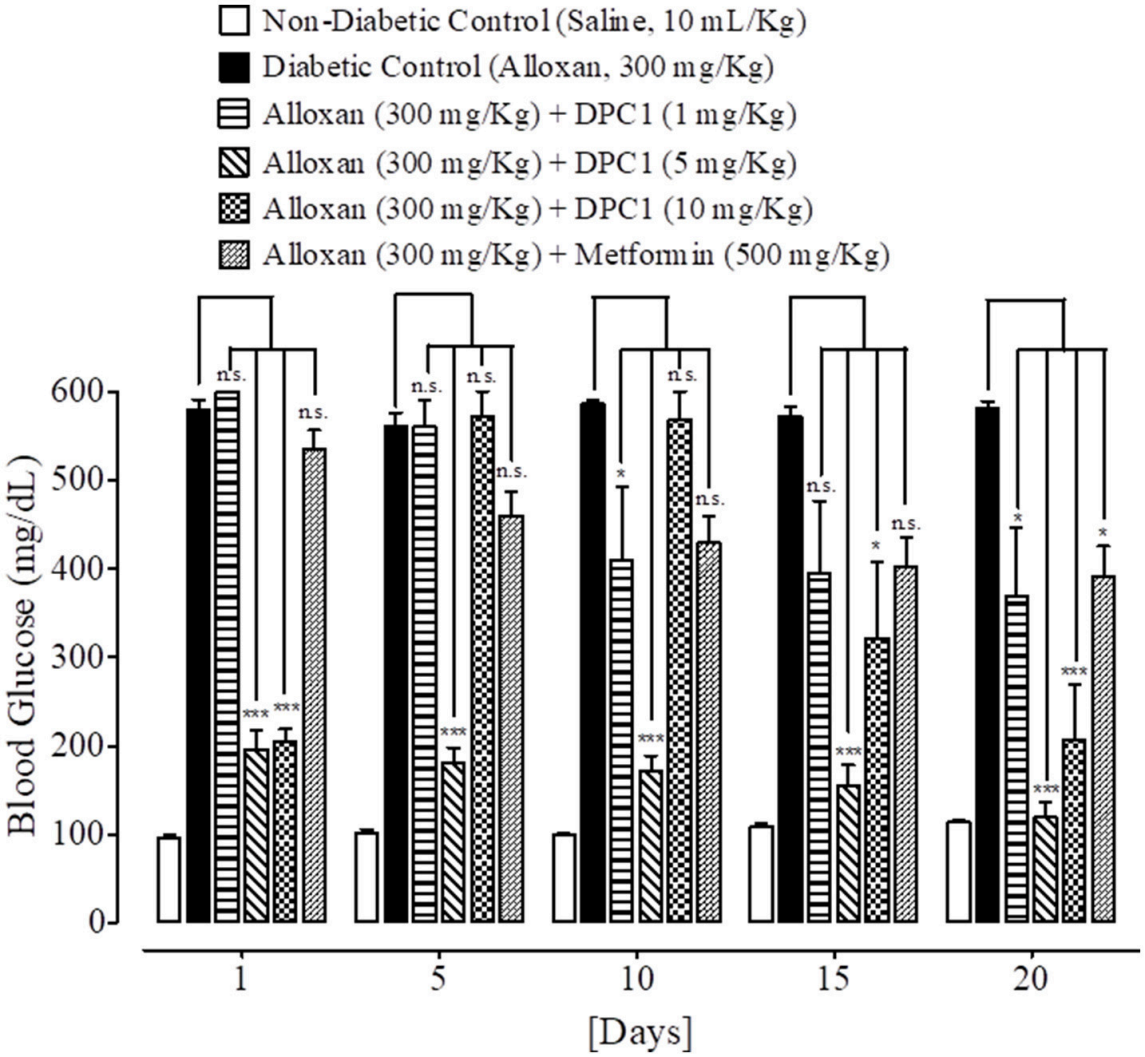
Bar-graph representing blood glucose levels at different treatment days of saline treated group (non-diabetic control), alloxan treated group (diabetic control), inhibitory effect of 1-(4-chlorobenzoyl)-3-(4-ferrocenylphenyl) urea (DPC1) at different doses (1–10 mg/Kg) and metformin treated group against alloxan-induced hyperglycemia in mice. Data presented as mean ± SEM. Statistical analysis used one-way ANOVA, followed by *post-hoc* Tukey's test. ^*^P < 0.05, ^***^P < 0.001 comparison of the blood glucose levels of DPC1 and metformin treated groups vs. diabetic control group. n.s., non-significant. The sample size in each group comprised of five mice (*n* = 5).

At day 1, 5, 10, 15, and 20th, blood glucose levels of non-diabetic control (saline, 10 mL/Kg) group were 96 ± 4.11, 102 ± 3.74, 99 ± 2.44, 109 ± 3.63, and 114 ± 3.20 mg/dL respectively. Blood glucose levels of alloxan (300 mg/Kg) treated diabetic control group were 578 ± 12.55, 560 ± 15.78, 586 ± 4.78, 572 ± 9.66, and 581 ± 7.94 mg/dL respectively. Blood glucose levels of DPAA (1 mg/Kg) treated group were 522 ± 19.70, 500 ± 24.59, 447 ± 17.48 (*P* < 0.05 vs. diabetic control), 470 ± 20.26 and 548 ± 29.25 mg/dL respectively. Blood glucose levels of DPAA (5 mg/Kg) treated group were 361 ± 60.93, 215 ± 65.25, 174 ± 47.36, 302 ± 28.13, and 318 ± 30.93 mg/dL (*P* < 0.001 vs. diabetic control) respectively. Blood glucose levels of DPAA (10 mg/Kg) treated group were 273 ± 37.69, 167 ± 40.54, 139 ± 31.11, 131 ± 30.78, and 102 ± 6.77 mg/dL (*P* < 0.001 vs. diabetic control) respectively. Blood glucose levels of metformin (500 mg/Kg) treated group were 534 ± 21.98, 460 ± 26.25, 429 ± 30.01 (*P* < 0.01 vs. diabetic control), 402 ± 32.67 and 391 ± 34.24 mg/dL (*P* < 0.001 vs. diabetic control) respectively (Figure 5). Blood glucose levels of DPC1 (1 mg/Kg) treated group were 600 ± 0.00, 560 ± 28.60, 409 ± 82.53, 395 ± 80.70, and 369 ± 76.86 mg/dL (*P* < 0.05 vs. diabetic control) respectively. Blood glucose levels of DPC1 (5 mg/Kg) treated group were 196 ± 21.91, 181 ± 17.08, 170 ± 18.25, 154 ± 24.03, and 119 ± 17.99 mg/dL (*P* < 0.001 vs. diabetic control) respectively. Blood glucose levels of DPC1 (10 mg/Kg) treated group were 204 ± 14.87 (*P* < 0.001 vs. diabetic control), 570 ± 29.20, 568 ± 31.60, 321 ± 86.36 (*P* < 0.05 vs. diabetic control), and 207 ± 61.84 mg/dL (*P* < 0.001 vs. diabetic control) respectively (Figure 6).

### Effect on body weight

At 20th treatment day, body weight of DPAA 1, 5 and 10 mg/Kg treated groups were improved by +3.8, +3.5, and +2.3 g values respectively. Body weight of metformin (500 mg/Kg) treated group was reduced to −1.8 g at 20th treatment day (Table 5). At 20th treatment day, body weight of DPC1 1, 5, and 10 mg/Kg treated group changed by −1.0, +5.0, and −0.6 g values respectively (Table 6).

**Table 5:** Effect of bis (diphenylacetato) cadmium (II) (DPAA) and metformin at different treatment days on body weight (g) of alloxan-induced diabetic mice.

**Treatment**	**Day 1**	**Day 5**	**Day 10**	**Day 15**	**Day 20**
Alloxan (300 mg/Kg) + DPAA (1 mg/Kg)	29.0 ± 2.09	30.5 ± 1.78	31.0 ± 1.54	32.4 ± 1.66	32.8 ± 1.62
Alloxan (300 mg/Kg) + DPAA (5 mg/Kg)	32.3 ± 1.07	33.0 ± 1.29	34.3 ± 1.25	35.3 ± 1.36	35.8 ± 1.36
Alloxan (300 mg/Kg) + DPAA (10 mg/Kg)	34.9 ± 0.85	35.8 ± 0.31	36.5 ± 0.70	36.9 ± 0.82	37.2 ± 0.89
Alloxan (300 mg/Kg) + Metformin (500 mg/Kg)	23.3 ± 1.22	22.7 ± 1.32	22.3 ± 1.33	21.7 ± 1.34	21.5 ± 1.35

*Data presented as mean ± SEM. The sample size in each group comprised of five animals (n = 5)*.

**Table 6:** Effect of 1-(4-chlorobenzoyl)-3-(4-ferrocenylphenyl) urea (DPC1) at different treatment days on body weight (g) of alloxan-induced diabetic mice.

**Treatment**	**Day 1**	**Day 5**	**Day 10**	**Day 15**	**Day 20**
Alloxan (300 mg/Kg) + DPC1 (1 mg/Kg)	32.9 ± 0.79	31.0 ± 1.48	29.9 ± 1.51	29.9 ± 1.36	31.9 ± 1.20
Alloxan (300 mg/Kg) + DPC1 (5 mg/Kg)	30.3 ± 1.94	30.0 ± 0.87	29.4 ± 0.89	32.9 ± 1.48	35.3 ± 1.27
Alloxan (300 mg/Kg) + DPC1 (10 mg/Kg)	34.5 ± 0.47	31.3 ± 1.00	30.4 ± 0.43	31.6 ± 1.34	33.9 ± 0.53

*Data presented as mean ± SEM. The sample size in each group comprised of five animals (n = 5)*.

### Effect on glucose tolerance

**Figure 7:**
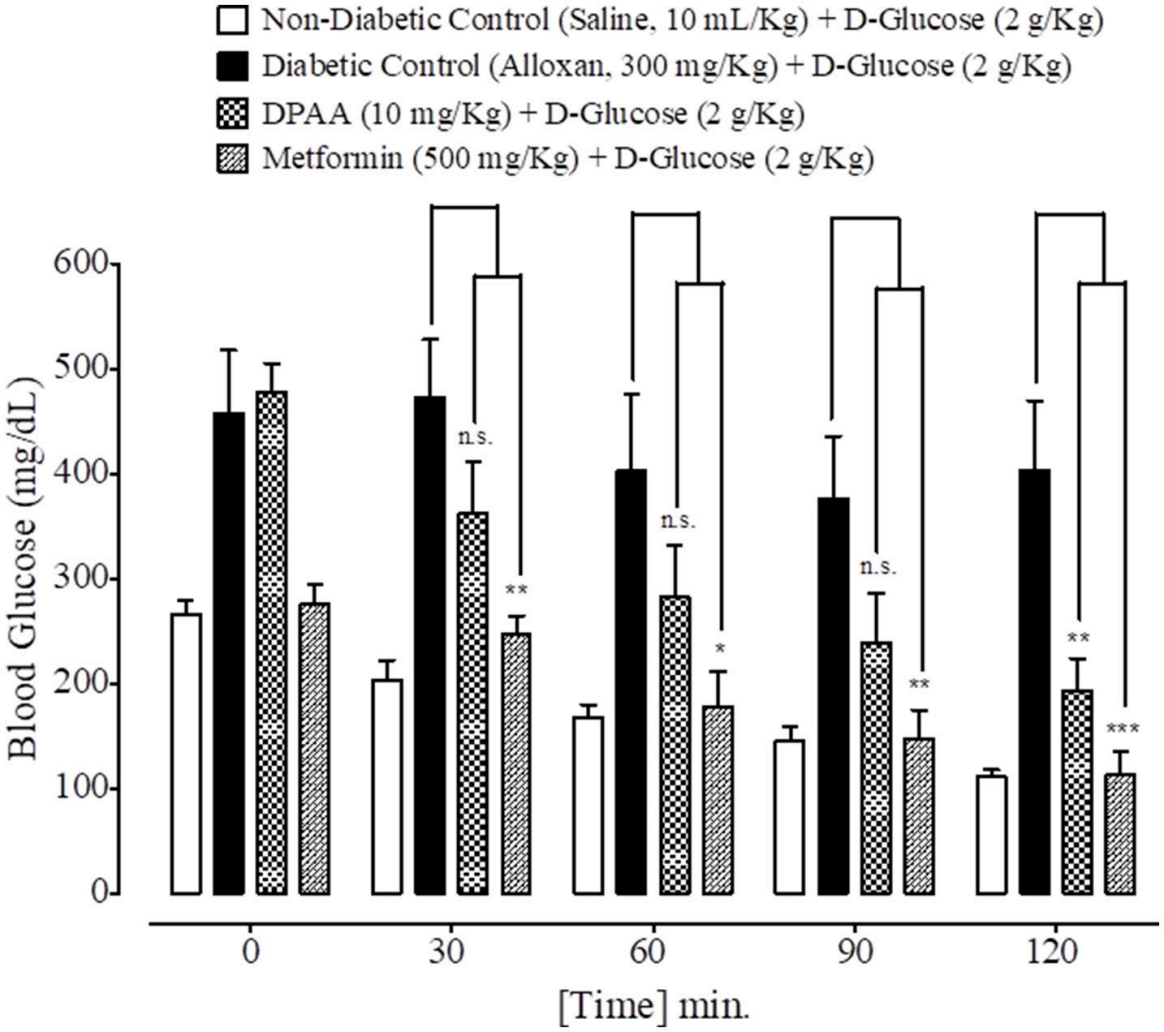
Bar graph representing blood glucose levels at different time intervals (0–120 min.) after administration of oral glucose load in mice of saline treated group (non-diabetic control), alloxan treated group (diabetic control), bis (diphenylacetato) cadmium (II) (DPAA) treated group and metformin pre-treated group. Data expressed as mean ± SEM. Statistical analysis used one-way ANOVA, followed by *post-hoc* Tukey's test. ^*^P < 0.05, ^**^P < 0.01, ^***^P < 0.001 comparison of the blood glucose levels of DPAA and metformin treated group vs. diabetic control group. n.s., non-significant. The sample size in each group comprised of five mice (*n* = 5).

**Figure 8:**
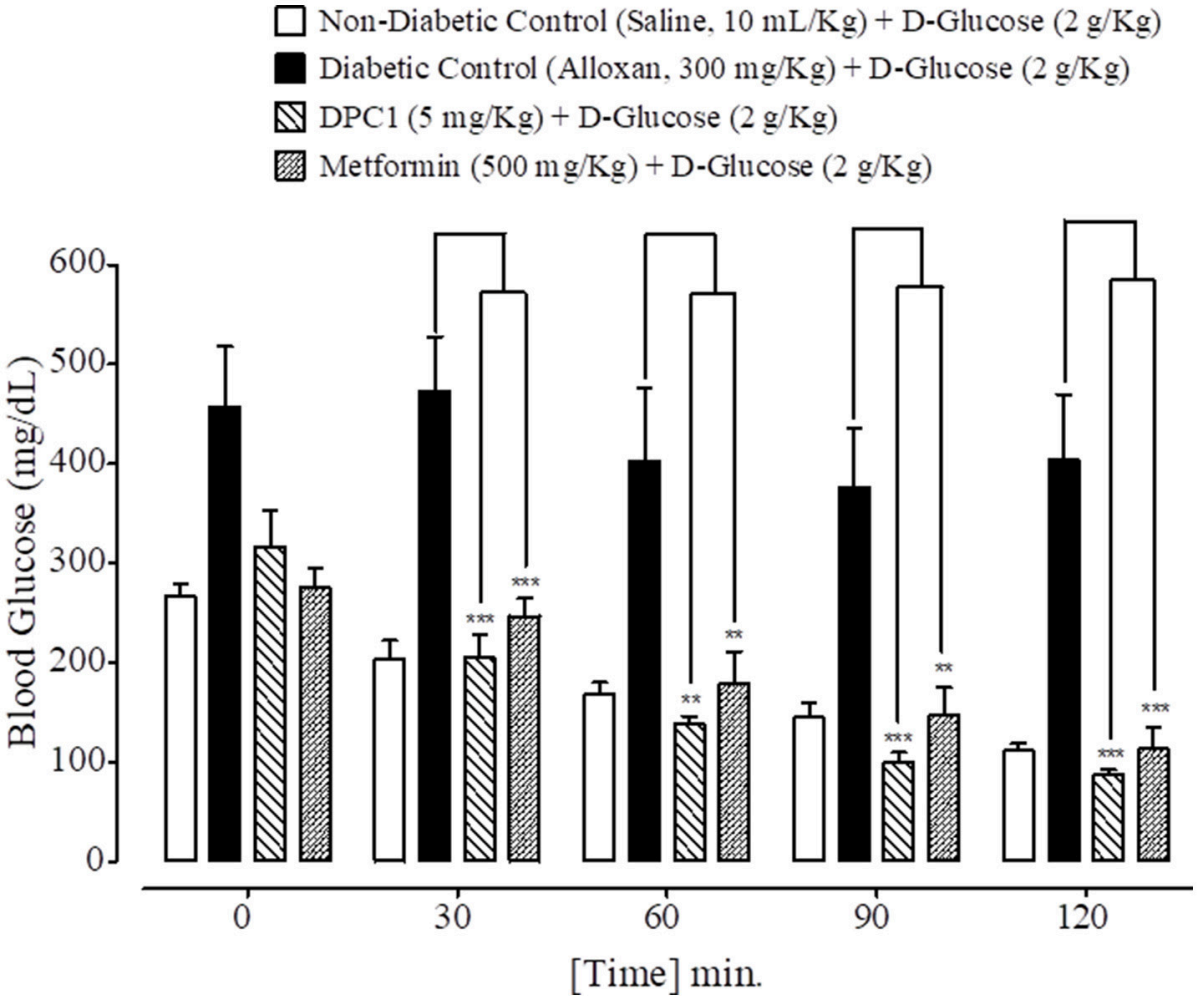
Bar graph representing blood glucose levels at different time intervals (0–120 min.) after administration of oral glucose load in mice of saline treated group (non-diabetic control), alloxan treated group (diabetic control), 1-(4-chlorobenzoyl)-3-(4-ferrocenylphenyl) urea (DPC1) treated group and metformin treated group. Data expressed as mean ± SEM. Statistical analysis used one-way ANOVA, followed by *post-hoc* Tukey's test. ^**^P < 0.01, ^***^P < 0.001 comparison of the blood glucose levels of DPC1 and metformin treated group vs. diabetic control group. The sample size in each group comprised of five mice (*n* = 5).

At 0, 30, 60, 90, and 120 min., blood glucose levels of non-diabetic control (saline, 10 mL/Kg) group were 266 ± 12.53, 204 ± 18.50, 168 ± 11.97, 145 ± 14.17, and 111 ± 6.68 mg/dL respectively. Blood glucose levels of alloxan (300 mg/Kg) treated diabetic control group were 457 ± 60.44, 473 ± 54.63, 402 ± 73.62, 376 ± 59.66, and 403 ± 66.36 mg/dL respectively. Blood glucose levels of DPAA (10 mg/Kg) treated group were 477 ± 28.40, 361 ± 50.34, 283 ± 48.48, 238 ± 46.97, and 193 ± 30.19 mg/dL (*P* < 0.001 vs. diabetic control) respectively. Blood glucose levels of metformin (500 mg/Kg) treated group were 275 ± 19.46, 246 ± 18.20 (*P* < 0.01 vs. diabetic control), 178 ± 32.80 (*P* < 0.05 vs. diabetic control), 147 ± 27.66 (*P* < 0.01 vs. diabetic control) and 113 ± 21.55 mg/dL (*P* < 0.001 vs. diabetic control) respectively (Figure 7). Blood glucose levels of DPC1 (5 mg/Kg) treated group were 317 ± 36.79, 205 ± 23.35, 138 ± 8.12, 100 ± 9.69, and 87 ± 4.97 mg/dL, with significance level of *P* < 0.001 vs. diabetic control at 30, 90, and 120 min., while *P* < 0.01 vs. diabetic control at 60 min (Figure 8).

### Effect on HbA1C

HbA1C value of non-diabetic control (saline, 10 mL/Kg) group was 3.1%. DPAA and DPC1 (1, 5, and 10 mg/Kg) treated group showed significant (*P* < 0.001 vs. diabetic control group) reduction in the HbA1C levels in alloxan-induced diabetic animals. Metformin (500 mg/Kg) treated showed reduction in HbA1C levels having *P* < 0.001 compared to the diabetic control group (Table 7).

**Table 7:** Effect of bis (diphenylacetato) cadmium (II) (DPAA), 1-(4-chlorobenzoyl)-3-(4-ferrocenylphenyl) urea (DPC1) and metformin on glycosylated hemoglobin A1C (HbA1C) in mice.

**Groups**	**HbA1C Levels (%)**
Non-Diabetic Control (Saline, 10 mL/Kg)	3.1 ± 0.05
Diabetic Control (Alloxan, 300 mg/Kg)	6.6 ± 0.11
Alloxan (300 mg/Kg) + DPAA (1 mg/Kg)	4.4 ± 0.10^***^
Alloxan (300 mg/Kg) + DPAA (5 mg/Kg)	3.6 ± 0.06^***^
Alloxan (300 mg/Kg) + DPAA (10 mg/Kg)	3.3 ± 0.12^***^
Alloxan (300 mg/Kg) + DPC1 (1 mg/Kg)	4.2 ± 0.07^***^
Alloxan (300 mg/Kg) + DPC1 (5 mg/Kg)	3.9 ± 0.05^***^
Alloxan (300 mg/Kg) + DPC1 (10 mg/Kg)	3.7 ± 0.18^***^
Alloxan (300 mg/Kg) + Metformin (500 mg/Kg)	3.4 ± 0.09^***^

Data expressed as mean ± SEM. Statistical analysis used one-way ANOVA, followed by post-hoc Tukey's test.

*** *P < 0.001 comparison of the HbA1C levels of DPAA, DPC1 and metformin treated groups vs. diabetic control group. The sample size in each group comprised of five mice (n = 5)*.

### Acute toxicity

DPAA at doses of 15, 25, 50, and 100 mg/Kg caused 40, 80, and 100% mortality respectively. DPC1 at tested doses of 25, 50, and 100 mg/Kg did not exhibit any mortality (Table 8).

**Table 8:** Percentage (%age) mortality of mice caused by bis (diphenylacetato) cadmium (II) (DPAA) and 1-(4-chlorobenzoyl)-3-(4-ferrocenylphenyl) urea (DPC1) at different doses.

**Test Compounds**	**Dose (mg/Kg)**	**Mortality (%)**
DPAA	15	40
	25	80
	50	100
	100	100
DPC1	25	0
	50	0
	100	0

*Mortality (%) = (No. of dead mice/Total No. of mice in group) × 100. The sample size in each group comprised of five mice (n = 5)*.

## Discussion

The application of computational approaches has turn out to be vital constituent of drug discovery strategy processes and ligand/structure based virtual screening is extensively used for this purpose (Langer and Hoffmann, 2001; Bajorath, 2002). From 1980s, molecular docking was found to be a key method of structure based virtual screening and it is still a very active area in research (Kuntz et al., 1982; Gohlke and Klebe, 2002; Kitchen et al., 2004). Virtual screening carried out through molecular docking that has become essential for quick and cost effective screening of the ligands on basis of structures (de Lange et al., 2014; Zhong et al., 2015). Patch dock server used in the study, assess ligand-protein complex by scoring on basis of appropriate geometry and atomic desolvation free energy (Schneidman-Duhovny et al., 2005). Lower ACE value indicates lower desolvation energy which is favorable for ligand-protein complex (Guo et al., 2012). In stated cases, strength of π-π interaction for stabilization of structural complex is comparable to the strength of hydrogen bonding (Blakaj et al., 2001). In ground state, loss of π-π interaction does not lead to affect the active-site conformation but results in 20–30 times reduction in the rate constant of chemical activity (Pecsi et al., 2010). Hydrophobic interactions can also enhance affinity of ligand against target protein (Patil et al., 2010). Evaluation of binding affinity between ligands and proteins complexes was done by assessing ACE value, H-bonds, π-π interaction and hydrophobic interactions.

We have found in this study, DPAA showed best binding score with lowest ACE value against GK and most of the target proteins than standard and other test compounds. We can anticipate that it has highest binding affinity against GK. The ligands order of affinity against GK was found as; DPAA > DPC1 > DMC1 > DOC1 > CPAA > PFA > metformin. The ligands order of affinity against AR was shown as; DOC1 > DPC1 > DPAA > DMC1 > CPAA > PFA > metformin. Test compounds that are high in order formed π-π bonds, hydrophobic bonds and H-bonds with GK and AR, while metformin and miglitol showed only H-bonding. Moreover all ligands interact with allosteric binding site of GK (Matschinsky et al., 2006; Min et al., 2017) and AR (Antony and Vijayan, 2015). The ligands order of affinity against AA was found as; DPAA > DPC1 > DMC1 > DOC1 > CPAA > PFA > miglitol. Compounds with high affinity did not show binding with TRP59, ASP197, and GLU233 which are reported as essential amino acid residue of AA (Piparo et al., 2008). Only DMC1 showed interaction against TRP59, but do not show highest binding affinity. The ligands order of affinity against FBP1 was shown as; DPAA > DMC1 > DOC1 > DPC1 > PFA > metformin > CPAA. Along with H-bonds and hydrophobic interactions, other interactions such as alky, π-alky and van der waals interactions are shown by test compounds with high affinity. Amino acids; PRO188, ARG49, ALA51, ALA189, and PRO100 are found to be important. The ligands order of affinity against PEPCK was found as; DMC1 > DPAA > DOC1 > DPC1 > CPAA > PFA > metformin. All ligands exhibited interactions with reported binding site of PEPCK (Katiyar et al., 2015). Moreover, H-bonding is found to be important for ligand-PEPCK complex.

The ligands order of affinity against GP was found as; DOC1 > DPAA > DMC1 > DPC1 > CPAA > metformin > PFA. DOC1, DPAA, and DMC1 showed interactions with ASP283, a conservative amino acid (Hudson et al., 1993) and ARG569 that is responsible for salt bridge interactions (Barford and Johnson, 1989). The ligands order of affinity against N-AG was found as; DPAA > 1 > DMC1 > DPC1 > CPAA > PFA > miglitol. Ligands are not involved in making any strong bonding with reported binding site (Saqib and Siddiqi, 2008). The ligands order of affinity against C-AG was found as; DPAA > DPC1 > DOC1 > DMC1 > CPAA > PFA > miglitol. H-bonds and hydrophobic interactions are found to be important, but ligands did not show bonding with stated binding site (Ren et al., 2011). Amino acid ASN1776 is found to be vital. The ligands order of affinity against GSK-3β was found as; DPAA > DPC1 > DOC1 > DMC1 > CPAA > TDZD-8 > PFA. All ferrocene derivatives showed interactions with CYS199 which is reported as important amino acid of binding site (Perez et al., 2011). DPAA lack interaction with CYS199, but still exhibited high binding affinity.

The ligands order of affinity against 11β-HSD1 was found as; carbenoxolone > DPAA > DPC1 > DMC1 > DOC1 > CPAA > PFA. Carbenoxolone, DPAA and DPC1 exhibited high affinity and formed interactions with TYR177 which is reported as key amino acid (Kim et al., 2006). The ligands order of affinity against p-Akt was found as; DPAA > DOC1 > rosiglitazone > DMC1 > DPC1 > PFA > CPAA. Ligands having high binding affinity formed interactions with TYR272 and VAL270. The ligands order of affinity against PI3K was found as; rosiglitazone > DPAA > CPAA > DOC1 > DPC1 > DMC1 > PFA. It is revealed that homoleptic cadmium carboxylates showed more affinity than ferrocene incorporated acyl ureas. The ligands order of affinity against PPAR-γ was found as; DPAA > rosiglitazone > DPC1 > DMC1 > DOC1 > CPAA > PFA. Ligand with high affinity showed hydrophobic interactions. All ligands showed interaction with ARG288, an essential amino acid of binding site (Choi et al., 2010). The ligands order of affinity against DPP IV was found as; DOC1 > DMC1 > DPC1 > DPAA > CPAA > sitagliptin > PFA. DOC1, DMC1 and DPC1 showed different interactions with HIS363, LEU410, and ALA409. These amino acid residues are found to be crucial against DPP IV. The ligands order of affinity against PTP-1B was found as; ertiprotafib > DPAA > DMC1 > DPC1 > CPAA > PFA > DOC1. Ligands showed interactions with amino acid PRO206. Ligands having high affinity showed H-bonds and hydrophobic interactions against PRO206. Interaction with amino acids of reported binding site was not shown by any ligand (Jin et al., 2016).

In current study, only enzymes are targeted that are involved in activation or inhibition of pathways important for pathogenesis of diabetes. By using molecular docking technique, ligands can be tested against other possible anti-diabetic targets such as sulfonylurea receptors, GLUT 1, GLUT 2, and GLUT 4 receptors as well as ion channels such as involvement of calcium channels, ligand gated K^+^ channels and Na^+^/K^+^ transporters. In result of virtual screening, DPAA and DPC1 are found to be potential agonists of GK. GK activating effect can be a proposed mechanism for anti-diabetic effect. Alloxan-induced diabetes model was used to validate the GK activating effect of DPAA and DPC1. It has been reported that GK activity was found to be same in alloxan- and streptozotocin-induced diabetes by depletion of β-cells as in control group (Matschinsky, 2009).

DPAA and DPC1 (1 mg/Kg) exhibited results like diabetic control group, so dose <1 mg/Kg cannot be used for significant anti-diabetic activity. DPAA (5 and 10 mg/Kg) and DPC1 (5 mg/Kg) showed time-dependent hypoglycemic effect than metformin. DPC1 (10 mg/Kg) produced abrupt increase in glucose levels at day 5 and 10th. Normally 1–2 mg of iron circulates in the blood (Andrews, 1999). Iron overload can lead to insulin resistance and impaired glucose utilization. Enhanced insulin sensitivity and glucose utilization has been reported in iron-deficient rats than iron-sufficient control group (Henderson et al., 1986; Borel et al., 1993). This effect can also be resulted by catalysis of highly reactive OH· radicals formation by iron via Fenton reaction (Crichton et al., 2002). DPC1 dose ≥10 mg/kg can reverse the hypoglycemic effect, while toxicity test revealed 10 mg/Kg as highest safest dose of DPAA.

DPAA and DPC1 reversed the reduced body weight compared to metformin. Both compounds enhanced the oral glucose tolerance as caused by metformin. Compounds produced dose-dependent effect in reducing HbA1C levels and found to be effective as long term anti-diabetic agent (Koenig et al., 1976). Enhanced hypoglycemic effect of DPAA could be due to the reduction of plasma selenium levels by cadmium moiety (Gümüşlü et al., 1997; Bleys et al., 2007) along with α-glucosidase inhibition by carboxylate group (Roy et al., 2015). Higher effect of DPC1 could be possible by GP inhibition due to acyl urea group (Klabunde et al., 2005). Antioxidant effect of DPC1 could also be the proposed mechanism for anti-diabetic activity (Asghar et al., 2015).

## Conclusions

Computational studies reveal binding affinities of selected ferrocene-based acyl ureas (PFA, DPC1, DMC1, and DOC1) and homoleptic cadmium carboxylates (DPAA and CPAA) against different proteins targets involved in pathogenesis of DM. Highest affinity was exhibited by DPAA and DPC1 against glucokinase. *In vivo* assays also validated the anti-diabetic effect of DPAA and DPC1. Both of the test compounds enhanced the glucose tolerance and decrease the HbA1C levels.

## Author contributions

SB carried out the computational studies, *in vivo* experimentations, evaluation of results and documentation. AK supervised the research project and drafted the final manuscript. FA and AB provided ferrocene derivatives. MU and SA provided the cadmium carboxylates. All authors read and approved the final manuscript.

### Conflict of interest statement

The authors declare that the research was conducted in the absence of any commercial or financial relationships that could be construed as a potential conflict of interest.
