# Supplementary material for: Computational and Pharmacological Evaluation of Ferrocene-Based Acyl Ureas and Homoleptic Cadmium Carboxylate Derivatives for Anti-diabetic Potential
Source: Front Pharmacol. 2018 Jan 17;8:1001. doi: 10.3389/fphar.2017.01001 (PMC5776112; doi:10.3389/fphar.2017.01001)
Supplement: Supplementary file 16 [file DataSheet1.DOCX]

**LEGENDS TO SUPPLEMENTARY (S) FIGURES**

**FIGURE** **1S.** **(A), (B), (C), (D), (E), (F)** and **(G)** represents 3D-interactions of ligands: 4-ferrocenyl aniline (PFA), 1-(4-chlorobenzoyl)-3-(4-ferrocenylphenyl) urea (DPC1), 1-(3-chlorobenzoyl)-3-(4-ferrocenylphenyl) urea (DMC1), 1-(2-chlorobenzoyl)-3-(4-ferrocenylphenyl) urea (DOC1), bis (diphenylacetato) cadmium (II) (DPAA), bis (4-chlorophenylacetato) cadmium (II) (CPAA) and miglitol with target: alpha amylase (AA) respectively, evaluated through Biovia Discovery Studio 2016.

**FIGURE** **2S.** **(A), (B), (C), (D), (E), (F)** and **(G)** represents 3D-interactions of ligands: 4-ferrocenyl aniline (PFA), 1-(4-chlorobenzoyl)-3-(4-ferrocenylphenyl) urea (DPC1), 1-(3-chlorobenzoyl)-3-(4-ferrocenylphenyl) urea (DMC1), 1-(2-chlorobenzoyl)-3-(4-ferrocenylphenyl) urea (DOC1), bis (diphenylacetato) cadmium (II) (DPAA), bis (4-chlorophenylacetato) cadmium (II) (CPAA) and miglitol with target: C-alpha glucosidase (C-AG) respectively, evaluated through Biovia Discovery Studio 2016.

**FIGURE** **3S.** **(A), (B), (C), (D), (E), (F)** and **(G)** represents 3D-interactions of ligands: 4-ferrocenyl aniline (PFA), 1-(4-chlorobenzoyl)-3-(4-ferrocenylphenyl) urea (DPC1), 1-(3-chlorobenzoyl)-3-(4-ferrocenylphenyl) urea (DMC1), 1-(2-chlorobenzoyl)-3-(4-ferrocenylphenyl) urea (DOC1), bis (diphenylacetato) cadmium (II) (DPAA), bis (4-chlorophenylacetato) cadmium (II) (CPAA) and miglitol with target: N-alpha glucosidase (N-AG) respectively, evaluated through Biovia Discovery Studio 2016.

**FIGURE** **4S.** **(A), (B), (C), (D), (E), (F)** and **(G)** represents 3D-interactions of ligands: 4-ferrocenyl aniline (PFA), 1-(4-chlorobenzoyl)-3-(4-ferrocenylphenyl) urea (DPC1), 1-(3-chlorobenzoyl)-3-(4-ferrocenylphenyl) urea (DMC1), 1-(2-chlorobenzoyl)-3-(4-ferrocenylphenyl) urea (DOC1), bis (diphenylacetato) cadmium (II) (DPAA), bis (4-chlorophenylacetato) cadmium (II) (CPAA) and metformin with target: aldose reductase (AR) respectively, evaluated through Biovia Discovery Studio 2016.

**FIGURE** **5S.** **(A), (B), (C), (D), (E), (F)** and **(G)** represents 3D-interactions of ligands: 4-ferrocenyl aniline (PFA), 1-(4-chlorobenzoyl)-3-(4-ferrocenylphenyl) urea (DPC1), 1-(3-chlorobenzoyl)-3-(4-ferrocenylphenyl) urea (DMC1), 1-(2-chlorobenzoyl)-3-(4-ferrocenylphenyl) urea (DOC1), bis (diphenylacetato) cadmium (II) (DPAA), bis (4-chlorophenylacetato) cadmium (II) (CPAA) and metformin with target: glucokinase (GK) respectively, evaluated through Biovia Discovery Studio 2016.

**FIGURE** **6S.** **(A), (B), (C), (D), (E), (F)** and **(G)** represents 3D-interactions of ligands: 4-ferrocenyl aniline (PFA), 1-(4-chlorobenzoyl)-3-(4-ferrocenylphenyl) urea (DPC1), 1-(3-chlorobenzoyl)-3-(4-ferrocenylphenyl) urea (DMC1), 1-(2-chlorobenzoyl)-3-(4-ferrocenylphenyl) urea (DOC1), bis (diphenylacetato) cadmium (II) (DPAA), bis (4-chlorophenylacetato) cadmium (II) (CPAA) and metformin with target: glycogen phosphorylase (GP) respectively, evaluated through Biovia Discovery Studio 2016.

**FIGURE** **7S.** **(A), (B), (C), (D), (E), (F)** and **(G)** represents 3D-interactions of ligands: 4-ferrocenyl aniline (PFA), 1-(4-chlorobenzoyl)-3-(4-ferrocenylphenyl) urea (DPC1), 1-(3-chlorobenzoyl)-3-(4-ferrocenylphenyl) urea (DMC1), 1-(2-chlorobenzoyl)-3-(4-ferrocenylphenyl) urea (DOC1), bis (diphenylacetato) cadmium (II) (DPAA), bis (4-chlorophenylacetato) cadmium (II) (CPAA) and metformin with target: fructose-1,6-bisphosphatase (FBP1) respectively, evaluated through Biovia Discovery Studio 2016.

**FIGURE** **8S.** **(A), (B), (C), (D), (E), (F)** and **(G)** represents 3D-interactions of ligands: 4-ferrocenyl aniline (PFA), 1-(4-chlorobenzoyl)-3-(4-ferrocenylphenyl) urea (DPC1), 1-(3-chlorobenzoyl)-3-(4-ferrocenylphenyl) urea (DMC1), 1-(2-chlorobenzoyl)-3-(4-ferrocenylphenyl) urea (DOC1), bis (diphenylacetato) cadmium (II) (DPAA), bis (4-chlorophenylacetato) cadmium (II) (CPAA) and metformin with target: phosphoenolpyruvate carboxykinase (PEPCK) respectively, evaluated through Biovia Discovery Studio 2016.

**FIGURE** **9S.** **(A), (B), (C), (D), (E), (F)** and **(G)** represents 3D-interactions of ligands: 4-ferrocenyl aniline (PFA), 1-(4-chlorobenzoyl)-3-(4-ferrocenylphenyl) urea (DPC1), 1-(3-chlorobenzoyl)-3-(4-ferrocenylphenyl) urea (DMC1), 1-(2-chlorobenzoyl)-3-(4-ferrocenylphenyl) urea (DOC1), bis (diphenylacetato) cadmium (II) (DPAA), bis (4-chlorophenylacetato) cadmium (II) (CPAA) and carbenoxolone with target: 11β-hydroxysteroid dehydrogenase-1 (11β-HSD1) respectively, evaluated through Biovia Discovery Studio 2016.

**FIGURE** **10S.** **(A), (B), (C), (D), (E), (F)** and **(G)** represents 3D-interactions of ligands: 4-ferrocenyl aniline (PFA), 1-(4-chlorobenzoyl)-3-(4-ferrocenylphenyl) urea (DPC1), 1-(3-chlorobenzoyl)-3-(4-ferrocenylphenyl) urea (DMC1), 1-(2-chlorobenzoyl)-3-(4-ferrocenylphenyl) urea (DOC1), bis (diphenylacetato) cadmium (II) (DPAA), bis (4-chlorophenylacetato) cadmium (II) (CPAA) and thiadiazolidinone-8 (TDZD-8) with target: glycogen synthase kinases-3β (GSK-3β) respectively, evaluated through Biovia Discovery Studio 2016.

**FIGURE** **11S.** **(A), (B), (C), (D), (E), (F)** and **(G)** represents 3D-interactions of ligands: 4-ferrocenyl aniline (PFA), 1-(4-chlorobenzoyl)-3-(4-ferrocenylphenyl) urea (DPC1), 1-(3-chlorobenzoyl)-3-(4-ferrocenylphenyl) urea (DMC1), 1-(2-chlorobenzoyl)-3-(4-ferrocenylphenyl) urea (DOC1), bis (diphenylacetato) cadmium (II) (DPAA), bis (4-chlorophenylacetato) cadmium (II) (CPAA) and rosiglitazone with target: peroxisome proliferator-activated receptor γ (PPAR-γ) respectively, evaluated through Biovia Discovery Studio 2016.

**FIGURE** **12S.** **(A), (B), (C), (D), (E), (F)** and **(G)** represents 3D-interactions of ligands: 4-ferrocenyl aniline (PFA), 1-(4-chlorobenzoyl)-3-(4-ferrocenylphenyl) urea (DPC1), 1-(3-chlorobenzoyl)-3-(4-ferrocenylphenyl) urea (DMC1), 1-(2-chlorobenzoyl)-3-(4-ferrocenylphenyl) urea (DOC1), bis (diphenylacetato) cadmium (II) (DPAA), bis (4-chlorophenylacetato) cadmium (II) (CPAA) and rosiglitazone with target: phosphatidylinositol 3 kinase (PI3K) respectively, evaluated through Biovia Discovery Studio 2016.

**FIGURE** **13S.** **(A), (B), (C), (D), (E), (F)** and **(G)** represents 3D-interactions of ligands: 4-ferrocenyl aniline (PFA), 1-(4-chlorobenzoyl)-3-(4-ferrocenylphenyl) urea (DPC1), 1-(3-chlorobenzoyl)-3-(4-ferrocenylphenyl) urea (DMC1), 1-(2-chlorobenzoyl)-3-(4-ferrocenylphenyl) urea (DOC1), bis (diphenylacetato) cadmium (II) (DPAA), bis (4-chlorophenylacetato) cadmium (II) (CPAA) and rosiglitazone with target: phosphorylated-Akt (p-Akt) respectively, evaluated through Biovia Discovery Studio 2016.

**FIGURE** **14S.** **(A), (B), (C), (D), (E), (F)** and **(G)** represents 3D-interactions of ligands: 4-ferrocenyl aniline (PFA), 1-(4-chlorobenzoyl)-3-(4-ferrocenylphenyl) urea (DPC1), 1-(3-chlorobenzoyl)-3-(4-ferrocenylphenyl) urea (DMC1), 1-(2-chlorobenzoyl)-3-(4-ferrocenylphenyl) urea (DOC1), bis (diphenylacetato) cadmium (II) (DPAA), bis (4-chlorophenylacetato) cadmium (II) (CPAA) and sitagliptin with target: dipeptidyl peptidase-IV (DPP IV) respectively, evaluated through Biovia Discovery Studio 2016.

**FIGURE** **15S.** **(A), (B), (C), (D), (E), (F)** and **(G)** represents 3D-interactions of ligands: 4-ferrocenyl aniline (PFA), 1-(4-chlorobenzoyl)-3-(4-ferrocenylphenyl) urea (DPC1), 1-(3-chlorobenzoyl)-3-(4-ferrocenylphenyl) urea (DMC1), 1-(2-chlorobenzoyl)-3-(4-ferrocenylphenyl) urea (DOC1), bis (diphenylacetato) cadmium (II) (DPAA), bis (4-chlorophenylacetato) cadmium (II) (CPAA) and ertiprotafib with target: protein tyrosine phosphatases 1B (PTP-1B) respectively, evaluated through Biovia Discovery Studio 2016.
